# A quantum chemical study of the mechanisms of olefin addition to group 9 transition metal dioxo compounds

**DOI:** 10.1186/s40064-016-2582-x

**Published:** 2016-06-24

**Authors:** Issahaku Ahmed, Richard Tia, Evans Adei

**Affiliations:** Computational and Theoretical Chemistry Laboratory, Department of Chemistry, Kwame Nkrumah University of Science and Technology, Kumasi, Ghana

**Keywords:** Olefin oxidation, Epoxidation, Transition metal, Computational, Mechanism

## Abstract

The mechanistic aspects of ethylene addition to MO_2_(CH_2_)(CH_3_) (M=Co, Rh, Ir) have been investigated with a Hartree–Fock/DFT hybrid functional at the MO6/LACVP* and B3LYP/LACVP* levels of theory to elucidate the reaction pathways on the singlet, doublet and triplet potential energy surfaces (PES). In the reaction of the IrO_2_CH_2_CH_3_ complex with ethylene, [3 + 2]_C,O_ addition is the most plausible pathway on the singlet PES, the [3 + 2]_O,O_ addition is the most favoured pathway on the doublet surface whiles the stepwise [1 + 1] addition involving the oxygen atom of the complex in the first step and the carbon atom of the complex in the second step is the most plausible pathway on the triplet PES. For the reaction of the RhO_2_(CH_2_)(CH_3_) complex, the [2 + 2]_Rh,O_ addition pathway is the most favoured on the singlet surface, the [2 + 2]_Rh,C_ is the most plausible pathway on the triplet PES and [3 + 2]_C,O_ is the most plausible on the doublet surface. For the reactions of the CoO_2_(CH_2_)(CH_3_) complex, the [1 + 2]_O_ addition is the most plausible on the singlet PES, [3 + 2]_C=Co=O_ cycloaddition to form the five–membered intermediate is the most preferred pathway on the doublet PES, whiles on the triplet PES the preferred pathway is the [3 + 2] addition across the O=Co=O bond of the metal complex. The reactions of olefins with the Co dioxo complex have lower activation barriers for the preferred [3 + 2] and [2 + 2] addition pathways as well as fewer side reactions than those of the rhodium and iridium systems. This could imply that the cobalt dioxo complexes can efficiently and selectively catalyze specific reactions in oxidation of olefins than Rh and Ir oxo complexes will do and therefore Co oxo complexes may be better catalysts for specific oxidation reactions of olefins than Rh and Ir complexes are. The activation barriers for the formation of the four—or five-membered metallacycle intermediates through [2 + 2] or [3 + 2] cyclo-addition are lower on the triplet PES than on the singlet PES for the formation of similar analogues. There are fewer competitive reaction pathways on the triplet surface than on the singlet PES. Also, cycloadditions that seem impossible on the singlet PES seem possible on the doublet and or triplet PESs, this is the case typically for the Rh and Co complexes, illustrating the importance of multiple spin states in organometallic reactions.

**Graphical Abstract:**
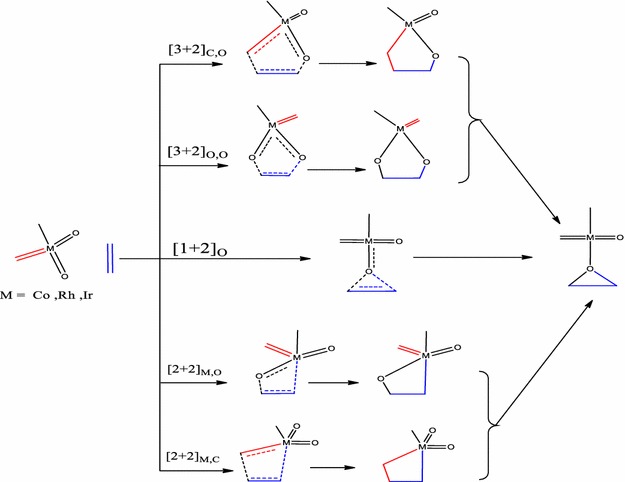
Table of Contents Synopsis: A study of the mechanism of ethylene addition to MO2(CH2)(CH3)(M=Co,Rh,Ir) shows the reactions of the Co complex have lower activation barriers for the preferred [3+2] and [2+2] addition pathways and fewer side reactions than those of Rh and Ir. Reactions are more feasible and selective on the triplet PES than on the singlet PES. These illustrate the importance of multiple spin states in organometallic reactions and shows catalyst activity and selectivity decreases down the group.

Table of Contents Synopsis: A study of the mechanism of ethylene addition to MO2(CH2)(CH3)(M=Co,Rh,Ir) shows the reactions of the Co complex have lower activation barriers for the preferred [3+2] and [2+2] addition pathways and fewer side reactions than those of Rh and Ir. Reactions are more feasible and selective on the triplet PES than on the singlet PES. These illustrate the importance of multiple spin states in organometallic reactions and shows catalyst activity and selectivity decreases down the group.

## Background

The oxidation of olefins with transition metal oxo compounds is an important class of oxygen transfer reactions (Kolb et al. 1994; Johnson and Sharpless 1993). One of the most useful reactions in which oxygen undergoes addition to an olefinic double bonds involves transition metal-oxo compounds and oxo-halides such as CrO_2_Cl_2_, OsO_4_, MnO_4_ (Enemark and Young 1993; Mijs and Jonge 1986; Sharpless et al. 1997; Sono et al. 1996). Transition metal-oxo complexes such as CrO_2_Cl_2_, OsO_2_(NH_2_), OsO_3_(CH_2_) react with olefins to form epoxides and metalladioxolanes (Tia and Adei 2011; Pidun et al. 1996; Haunschild and Frenking 2008a). Many experimental and theoretical investigations (Enemark and Young 1993; Mijs and Jonge 1986; Sharpless et al. 1997; Sono et al. 1996; Tia and Adei 2011; Pidun et al. 1996; Haunschild and Frenking 2008a; Criegee 1936; Criegee et al. 1942; Houk and Strassner 1999; Del Monte et al. 1997; Rouhi 1997) have focused on mechanistic aspects of this type of reactions.

Pidun et al. (1996) found that the initial step of the oxidation of olefins by OsO_4_ is a concerted [3 + 2] addition yielding an osma-2,5-dioxolane as reaction product. The activation barrier of this reaction pathway was significantly lower compared to the stepwise pathway which first involves a [2 + 2] addition of the olefin to the OsO_4_ yielding an osmaoxetane intermediate followed by rearrangement. Criegee (1936) and Criegee et al. (1942) proposed that the initial step of the dihydroxylation reaction of olefins catalyzed by transition metal oxo compound MnO_4_^−^ favoured a [2 + 3] addition to form a metalladioxolane, a five-membered metallacycle. Sharpless et al. (1997). suggested a stepwise mechanism involving a metallaoxetane intermediate in the chromyl chloride oxidation arising from a [2 + 2] addition followed by rearrangement to form the five-membered product. However, the intermediacy of a metallaoxetane arising from a [2 + 2] addition pathway as suggested by Sharpless et al. (1997) for chromyl chloride oxidation was ruled out, at least for MnO_4_^−^, by density functional theory (DFT) calculations (Houk and Strassner 1999) and corroborated by experimental kinetic isotope effects studies (Del Monte et al. 1997; Rouhi 1997).

Quantum chemical studies by Strassner and Busold (2001) on the oxidation of olefins by permanganate employing density functional theory at the B3LYP/6-31G(d) level showed the [3 + 2] addition to be favoured over the [2 + 2] addition. It has been found that when a transition metal–carbon double bond is present the reactivity trend changes. The [2 + 2] addition across the Os–CH_2_ double bond becomes competitive for the ethylene addition to OsO_3_(CH_2_) and OsO_2_(CH_2_)_2_ (Hölscher et al. 2005; Haunschild et al. 2007). For ReO_2_(CH_3_)(CH_2_) and WO(CH_3_)_2_(CH_2_), the [2 + 2] pathway across the carbon—metal double bond becomes even more favourable (Haunschild et al. 2007).

Density functional theory studies of the mechanisms of oxidation of ethylene by chromyl chloride had also been studied (Tia and Adei 2009; Torrent et al. 1999) as well as the oxidation of ethylene by the group VII transition metal-oxo complexes of the type LMO_3_ (M=Mn, Tc, Re and L=O–, Cl, CH_3_, OCH_3_, Cp) (Aniayei et al. 2013a, b, c). All these studies indicated that the [3 + 2] addition has a lower barrier than the [2 + 2] addition step in most cases on the singlet potential surface, but [2 + 2] addition was favoured in some cases especially on other PESs.

Haunschild and Frenking (2008a) carried out quantum chemical investigations on the reaction pathways for ethylene addition to IrO_2_(CH_3_)(CH_2_), RhO_2_(CH_3_)(CH_2_) and CoO_2_(CH_3_)(CH_2_). These investigations were limited to complexes in the singlet electronic state. The investigation showed that all the three complexes prefer a [3 + 2] cycloaddition pathway rather than a [2 + 2] addition. The iridium and rhodium complexes preferred the [3 + 2]_C,O_ pathway where the transition metal–carbon and transition metal–oxygen double bonds were involved whereas the cobalt complexes showed preference for the [3 + 2]_O,O_ addition where both transition metal–oxygen double bonds are participating with concomitant hydrogen migration. Earlier works by Hölscher et al. (2005), Haunschild et al. (2007), Cappel et al. (2006), Haunschild and Frenking (2007, 2008b) involving ethylene addition to ReO_2_(CH_3_)(CH_2_), WO(CH_3_)_2_(CH_2_), MoO(CH_3_)_2_(CH_2_), CrO(CH_3_)_2_(CH_2_) and TcO_2_(CH_3_)(CH_2_) indicates that the [2 + 2] pathway across the carbon metal double bond is the most favoured addition pathway.

Even though several works have been carried out on reactions of transition metal oxo complexes, there are still several unresolved mechanistic questions surrounding the reactions of transition metal oxo complexes with alkenes. Some of these unresolved questions include the oxidation of olefins by group-9 transition metal dioxo complexes on the doublet and triplet PESs. A change of spin state can affect the molecular structure of a complex in terms of bond lengths, angular distortions and the overall molecular geometry of the complex. Spin-crossing effects can intensely affect mechanisms of reactions, rate constants as well as temperature behaviors of organometallic conversion. Multiple surface participation (other than only singlet surface) pathways are proposed as key features in the chemistry of organometallic complexes. However, for gas phase reactions concerning organometallics, multiple spin surfaces connect reactants to products. This can provide low-energy paths for otherwise difficult processes (Buchachenko 2000; Schröder et al. 2000; Poli 1996; Harvey et al. 2003). It is therefore anticipated that spin states might play a key role in resolving some of the unsolved mechanistic questions which still surround the reaction of transition metal oxo complexes with alkenes.

**Scheme 1:**
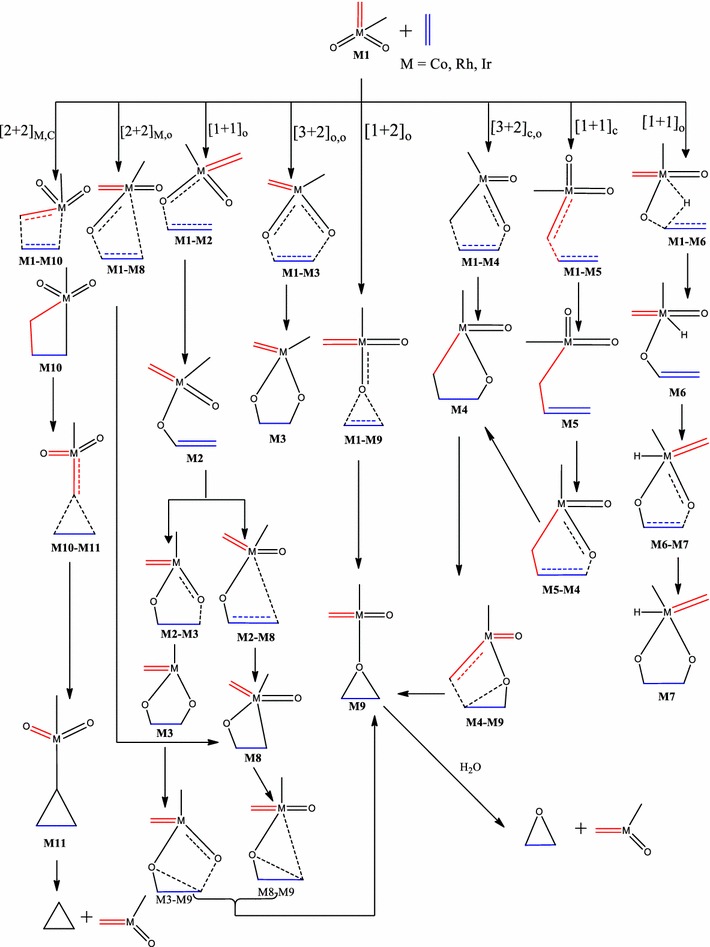
Proposed pathways for the reactions of MO_2_(CH_3_)(CH_2_) (M=Co, Rh, Ir) with ethylene

This work therefore seeks to extend the work of Haunschild and Frenking (2008a) by investigating the role multiple spin states play in the ethylene addition to CoO_2_(CH_3_)(CH_2_), RhO_2_(CH_3_)(CH_2_) and IrO_2_(CH_3_)(CH_2_) and provide possible reaction pathways so as to allow comparison of the reaction mechanism on the singlet, doublet and triplet electronic states. The mechanistic pathways to the formation of the epoxide precursor or epoxide are also explored. Other [3 + 2] and [2 + 2] addition pathways have been explored in addition to those already proposed and studied (Scheme 1).

## Computational details

We performed all the computations with the Spartan (2010) computational chemistry package developed by Wavefunction, Inc, versions 2008V1.2.0 and 2010V1.2.0, using the Becke-three-parameter Lee–Yang–Parr (B3LYP) hybrid exchange–correlational functional and the MO6 hybrid functional. The B3LYP functional, a Hartree–Fock DFT hybrid functional, is made up of the exchange–correlation energy from the local spin-density approximation (LSDA) method, 20 % of the difference between the Hartree–Fock exchange energy (Kohn–Sham exchange energy) and the LSDA exchange energy, 72 % of the Becke exchange potential (which includes the 1988 correction) (Becke 1988, 1996), 81 % of the Lee–Yang–Parr correlation potential (Lee et al. 1988) and 19 % of the Vosko-Wilk-Nusair potential (Vosko et al. 1980) and is one of the most widely used exchange-correlation functionals in organometallic chemistry. The MO6 functional (Zhao and Truhlar 2008) is a global hybrid meta-generalized gradient approximation (meta-GGA) with 27 % of Hartree–Fock exchange, leading to a well-balanced functional for overall good performance for chemistry. It has thus been recommended for application in organometallic and inorganometallic chemistry (Peverati and Truhlar 2014). The atoms hydrogen-chlorine were described with the 6-31G (d) basis set while the metal Re was described with the LANL2DZ basis set (Dunning TH Jr and Hay 1976; Hay and Wadt 1985a, b; Wadt and Hay 1985; Roy et al. 2008). The MO6/LANL2DZ and B3LYP/LANL2DZ are the two most popular DFT levels of theory for organometallic and inorganic chemistry to date. The latest review on DFT methods for computational studies of synthetically relevant homogeneous organometallic catalysis involving Ni, Pd, Ir, and Rh (Sperger et al. 2015) indicates that between the period 2009–2014 geometry optimization calculations are dominated by the LANL2DZ as ECP for the transition metal. Table 1d of that review shows that for the period 2013–2014, 51 % of all reviewed studies employed the LAN2DZ basis set for geometry optimization. It also indicates that currently, the first choice of functional for energy calculations of TM systems is MO6, followed by B3LYP, DFT-D3, and MO6L, whereas the choice of basis set for the description of the transition metal is dominated by LANL2DZ and SDD (the Stuttgart-Dresden ECP with double zeta basis set).

**Table 1:** Comparison of the peri-selectivity of the reactions of Ir, Rh and Co: activation barriers and reaction energies for the first step of the various reactions of the Ir, Rh and Co complexes with ethylene

Metal	MO_2_(CH_2_)(CH_3_)		Activation energy		Reaction energy	
	Reaction path	Addition	B3LYP	MO6	B3LYP	MO6
Singlet PES						
Ir	Ir1 Ir2	[1 + 1]	38.8 (39.40)	18.96 (19.21)	−20.67 (−20.10)	−24.88 (−23.76)
	Ir1 Ir3	[3 + 2]_O,O_	15.82 (16.23)	8.24 (8.88)	−42.50 (−41.28)	−49.65 (−48.19)
	Ir1 Ir4	[3 + 2]_C,O_	0.68 (0.89)	0.39 (0.52)	−35.85 (−33.94)	−38.84 (−36.66)
	Ir1 Ir8	[2 + 2]_Ir=O_	24.66 (25.52)	11.98 (12.29)	−20.87 (−19.16)	−25.09 (−24.84)
	Ir1 Ir10	[2 + 2]_Ir=C_	0.84 (1.01)	0.51 (0.72)	−18.89 (−18.20)	−21.43 (−20.97)
Rh	Rh1 Rh2	[1 + 1]	7.59 (8.43)	4.08 (4.76)	−21.90 (−21.34)	−23.56 (−23.11)
	Rh1 Rh8	[2 + 2]_Rh=O_	10.28 (10.97)	5.99 (6.84)	−26.30 (−25.59)	−29.59 (−28.68)
	Rh1 Rh9	[1 + 2]_O_	33.73 (34.08)	15.54 (16.56)	−5.42 (−4.97)	−8.45 (−7.91)
Co	Co1 Co2	[1 + 1]	7.19 (7.65)	3.98 (4.02)	−43.23 (−41.41)	−47.24 (−23.11)
	Co1 Co9	[1 + 2]_O_	4.95 (5.85)	2.49 (2.84)	−43.35 (−42.06)	−48.05 (−28.68)
Doublet PES						
Ir	Ir1 Ir2	[1 + 1]	20.27 (20.96)	11.12 (10.98)	−12.86 (−12.23)	−15.43 (−14.79)
	Ir1 Ir3	[3 + 2]_O,O_	26.67 (27.21)	12.89 (13.27)	−40.58 (−38.91)	−46.65 (−45.76)
	Ir1 Ir4	[3 + 2]_C,O_	8.81 (9.14)	4.21 (4.89)	−52.40 (−51.08)	−55.80 (−54.68)
	Ir1 Ir8	[2 + 2]_Ir=O_	30.49 (30.87)	16.38 (17.12)	9.43 (9.89)	7.61 (7.89)
Rh	Rh1 Rh2	[1 + 1]	13.79 (14.08)	7.12 (7.59)	−14.65 (−14.01)	−18.42 (−17.92)
	Rh1 Rh4	[3 + 2]_C,O_	3.17 (4.02)	2.04 (2.88)	−75.54 (−73.78)	−80.60 (−78.85)
	Rh1 Rh8	[2 + 2]_Rh=O_	25.92 (27.45)	13.78 (14.41)	−8.87 (−7.97)	−11.26 (−10.05)
	Rh1 Rh9	[1 + 2]_O_	14.49 (14.89)	6.92 (7.78)	−0.004 (0.07)	−0.02 (0.04)
Co	Co1 Co2	[1 + 1]	37.16 (38.32)	16.48 (17.05)	−80.50 (−79.84)	−89.12 (−88.63)
	Co1 Co3	[3 + 2]_O,O_	12.54 (13.02)	7.76 (7.39)	−56.40 (−54.98)	−58.96 (−58.01)
	Co1 Co4	[3 + 2]_O,C_	0.98 (1.25)	0.56 (0.87)	−4.82 (−19.38)	−6.98 (−5.77)
Triplet PES						
Ir	Ir1 Ir2	[1 + 1]	14.61 (15.24)	7.77 (8.22)	−17.09 (−16.94)	−20.32 (−20.21)
	Ir1 Ir5	[3 + 2]_O,O_	4.91 (5.61)	2.85 (3.45)	−18.84 (−18.08)	−22.41 (−21.97)
Rh	Rh1 Rh2	[1 + 1]	12.53 (12.94)	7.26 (7.99)	−14.56 (−14.04)	−17.47 (−18.21)
	Rh1 Rh5	[1 + 1]	1.98 (2.05)	1.02 (1.54)	−25.39 (−24.47)	−28.82 (−28.22)
	Rh1 Rh10	[2 + 2]_Rh=C_	11.98 (12.23)	6.31 (6.79)	−19.89 (−19.38)	−22.18 (−21.78)
Co	Co1 Co2	[1 + 1]	10.52 (10.87)	5.34 (5.98)	−5.72 (−4.47)	−7.72 (−7.13)
	Co1 Co3	[3 + 2]_O,O_	1.05 (1.76)	0.68 (0.89)	−46.80 (−46.14)	−52.89 (−51.52)

Zero-point corrected energies in parentheses. Energies are in kcal/mol

Spartan uses a graphical model builder for input preparation. Molecules were constructed and minimized interactively using an appropriate molecular mechanics force field. All structural optimizations were done without symmetry restrictions. Normal mode analysis was performed to verify the nature of the stationary points located. Minima, representing reactants, intermediates and products were shown to have no imaginary frequencies.

Guess structures for transition state calculations were obtained by first constraining specific bonds along the reaction coordinates at fixed lengths while the remaining internal coordinates were fully optimized. This procedure gives an approximate transition state guess which is then submitted for transition state calculation using the standard transition state optimization procedure in Spartan. All transition state structures were subjected to full normal mode analyses to ensure that they have a Hessian matrix with a single negative eigen-value, characterized by an imaginary vibrational frequency along the reaction coordinate. An intrinsic reaction coordinate (IRC) calculation was carried out to ensure that transition states smoothly connect reactants and products.

## Results and discussion

### Reaction between IrO_2_(CH_2_)(CH_3_) and ethylene

**Fig. 1:**
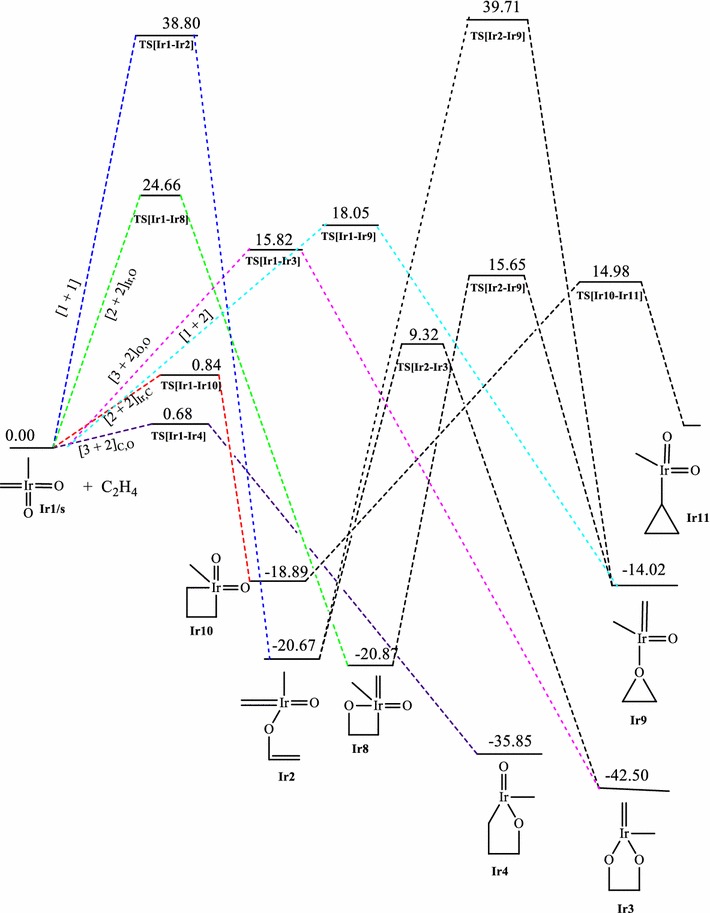
Energy profile of the reaction of IrO_2_(CH_2_)(CH_3_) with ethylene on the singlet PES at the B3LYP level of theory. Energies are in kcal/mol

**Fig. 2:**
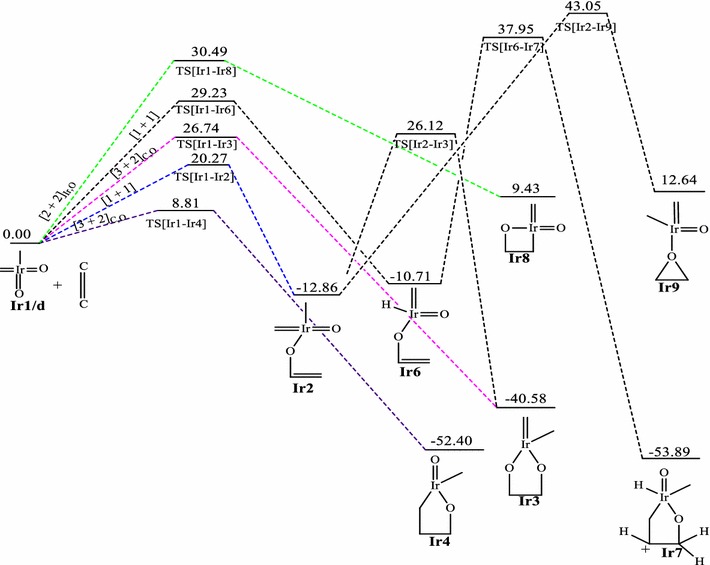
Energy profile of the reaction of IrO_2_(CH_2_)(CH_3_) with ethylene on the doublet PES at the B3LYP level of theory. Energies are in kcal/mol

**Fig. 3:**
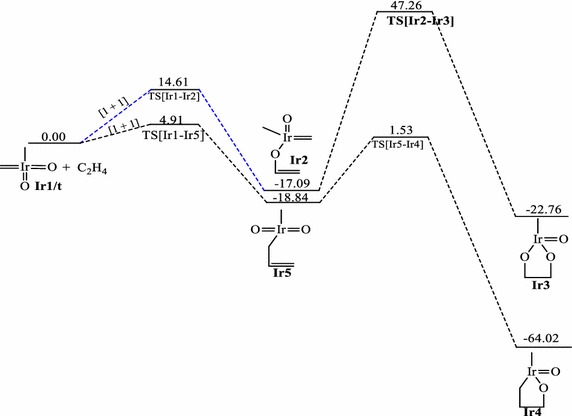
Energy profile of the reaction of IrO_2_(CH_2_)(CH_3_) with ethylene on the triplet PES at the B3LYP level of theory. Energies are in kcal/mol

The energetics of the reactions of IrO_2_CH_2_CH_3_ with ethylene on the singlet, doublet and triplet surfaces at the B3LYP level are shown on Figs. 1, 2, and 3 respectively. Table 1 gives a summary of the energetics of all the first steps of the reactions of MO_2_CH_2_CH_3_ (M=Ir, Rh, Co) at the MO6 and B3LYP levels of theory including zero-point energy corrections. The optimized geometries of all stationary points are included in the supplementary information. A triplet IrO_2_CH_2_CH_3_ reactant (**Ir1/t**) has been computed to be 4.72 kcal/mol more stable in relation to the singlet structure **Ir1/s.** All singlet and triplet structures were computed as neutral molecules whiles the doublet structures were computed as anionic species.

The activation barrier for the [3 + 2]_C,O_ addition to form the five membered metallaoxetane **Ir4** (Fig. 1) is 0.68 kcal/mol and the reaction energy is −35.50 kcal/mol on the singlet PES at the B3LYP level of theory. Formation of the same product on the triplet PES (Fig. 3) has an activation barrier of 25.28 kcal/mol and relative reaction energy of −64.02 kcal/mol. Haunschild and Frenking (2008a) computed activation barrier and relative energy of 50.00 and 5.30 kcal/mol respectively on the singlet PES at the B3YLYP level of theory. Even though the path of lower barrier for the formation of the five-membered metallaoxetane **Ir4** is on the singlet surface (Fig. 1), the product is more stable on the triplet surface (Fig. 3). Therefore the most likely reaction route is from singlet reactant through singlet transition state to triplet metallaoxetane **Ir4**, a case of two-state reactivity as illustrated in scheme 3b of the seminal work of Schröder et al. (2000) on two-state reactivity. The addition through [3 + 2]_O,O_ to form the five-membered dioxylate has a much higher barrier of 15.82 kcal/mol and reaction energy of −42.50 kcal/mol on the singlet PES at the B3LYP level of theory. Formation of the same product on the triplet PES has activation barrier of 78.96 kcal/mol with reaction energy of −22.76 kcal/mol at the B3LYP level of theory. However, [2 + 2] cycloaddition across Ir=CH_2_ leading to **Ir10** on the singlet PES has a barrier of 0.84 kcal/mol which is lower than the barrier for the [3 + 2]_O,O_ and, addition across Ir=O leading to **Ir8** has a barrier of 24.66 kcal/mol which is higher than the barrier for the [3 + 2]_O,O_. A transition state for direct addition was not located for the formation of the five-membered metallaoxetane and the dioxylate on the triplet PES, but a two-step mechanism through a stepwise [1 + 1] addition involving the oxygen atom of the complex in the first step and the carbon atom of the complex in the second step to form the metallaoxetane and a stepwise [1 + 1]_O,O_ addition between one oxygen of the complex with one carbon of ethylene to form the dioxylate. The species **Ir9** (epoxide precursor) was found to have come from direct side-on attack of C_2_H_4_ on one oxygen atom of **Ir1** which is an intermediate for the formation of the four membered metallaoxetane **Ir8**. However, epoxide precursor **Ir9** in principle could arise from five pathways:

- A two-step process involving [2 + 2] addition of C_2_H_4_ across the Ir=O bond of **Ir1**
- A one-step direct addition of ethylene to one oxygen atom of **Ir1**
- A two-step process involving [3 + 2] addition of ethylene across the two oxygen atoms of **Ir1**
- A two-step process involving [3 + 2] addition of ethylene across the oxygen and the carbon double bond of **Ir1**
- A two-step process involving addition of one carbon of the C_2_H_4_ to one oxygen of **Ir1** followed by the other carbon of C_2_H_4_ with the same oxygen atom.

Four of these pathways, none of which has been reported to date, were located on the singlet PES, one was located on the doublet surface whiles none was located on the triplet surface. The most favoured pathway towards the formation of the epoxide is the [1 + 2]_O_ addition on the singlet surface which has an energy barrier of 18.05 kcal/mol. The dioxylate analogue species **Ir7** (Fig. 2) located only on the doublet surface was formed through addition of the carbon atom of the ethylene ligand and the one of the oxygen atoms of the iridium complex, followed by rearrangement of the intermediate to form the dioxylate analogue with hydrogen atom of the carbon atom migrating to the metal atom. The formation of the four-membered metallaoxetane and dioxylane are more favourable on the singlet surface than on the doublet and triplet surfaces.

The triplet PES did not give the usual [2 + 2] addition either across the metal–carbon double bond or across the metal–oxygen double bond. It was found that for the reaction of the IrO_2_(CH_2_)(CH_3_) complex, the [3 + 2]_C,O_ addition is the most plausible pathway on the singlet PES consistent with the work of Haunschild and Frenking (2008a), the [3 + 2]_O,O_ is the most favoured pathway on the doublet surface whiles the stepwise [1 + 1] addition involving the oxygen atom of the complex in the first step and the carbon atom of the complex in the second step is the most plausible pathway on the triplet PES. The possibility and plausibility of the reaction of IrO_2_(CH_2_)(CH_3_) on the doublet and triplet PES were not reported in the earlier works of Haunschild and Frenking (2008a).

The order of the energetically most favorable addition reaction for the iridium system on the singlet potential energy surface is found to be in the order [3 + 2]_C,O_ > [2 + 2]_Ir,C_ > [3 + 2]_O,O_ > [2 + 2]_Ir,O_ > s[1 + 1]_O,O_. That on the doublet surface is [3 + 2]_C,O_ > [3 + 2]_O,O_ > [2 + 2]_Ir,O_ and the triplet surface is in the order of s[1 + 1]_C,O_ > s[1 + 1]_O,O_.

The calculated activation and reaction energies for addition pathways (Table 1) at the MO6 level of theory are lower than those at the B3LYP, which is consistent with the work of Linder and Brinck (2012) and Ahmed et al. (2015a, b) but both levels predict the same preferred addition pathways and the same trends.

### Reaction between RhO_2_(CH_2_)(CH_3_) and ethylene

**Fig. 4:**
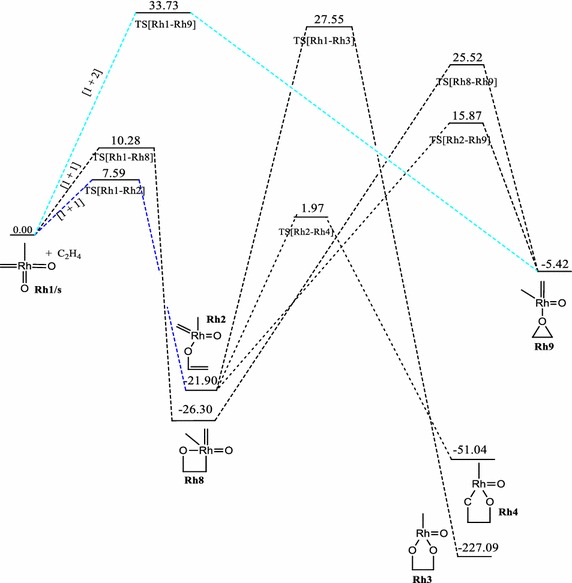
Energy profile of the reaction of RhO_2_(CH_2_)(CH_3_) with ethylene on the singlet PES at the B3LYP level of theory. Energies are in kcal/mol

**Fig. 5:**
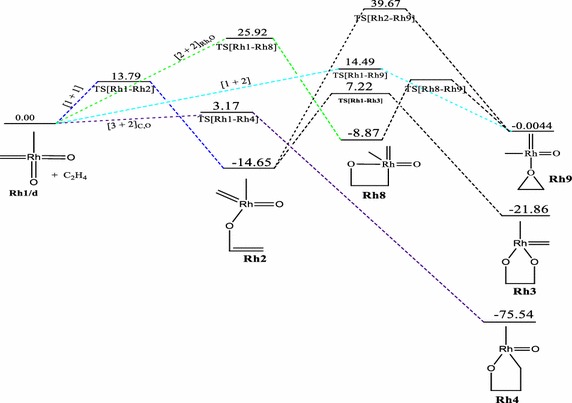
Energy profile of the reaction of RhO_2_(CH_2_)(CH_3_) with ethylene on the doublet PES at the B3LYP level of theory. Energies are in kcal/mol

**Fig. 6:**
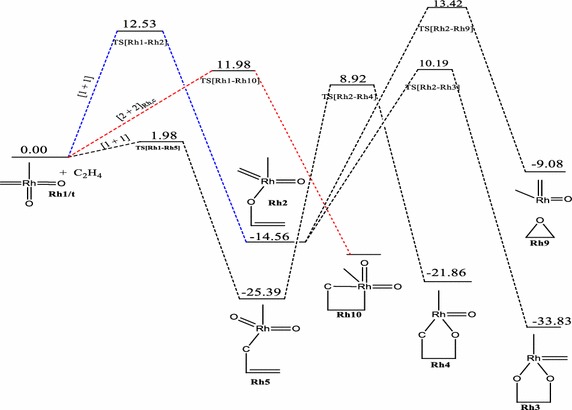
Energy profile of the reaction of RhO_2_(CH_2_)(CH_3_) with ethylene on the triplet PES at the B3LYP level of theory. Energies are in kcal/mol

The relative energies of the main stationary points involved in the reaction between RhO_2_CH_2_CH_3_ and ethylene on the singlet, doublet and triplet surfaces are shown on Figs. 4, 5 and 6 respectively. A triplet RhO_2_(CH_2_)(CH_3_) reactant (**Rh1/t**) has been computed to be 4.55 kcal/mol more stable in relation to the singlet structure **Rh1/s**. All singlet and triplet structures were computed as neutral molecules whiles the doublet structures were computed as anionic species.

Just like the iridium system, a [3 + 2], [2 + 2] and [1 + 2] cycloaddition reactions have been found for the **Rh1** + C_2_H_4_ system. The addition through the [3 + 2]_C,O_ in the formation of the five membered metallaoxetane (**Rh4)** analogue Fig. 5 is highly exothermic by 75.54 kcal/mol on the doublet PES and has a very low barrier of 3.17 kcal/mol. The doublet PES is both thermodynamically and kinetically most favoured. From Figs. 4 and 6, it could be seen that the formation of five-membered metallaoxetane analogue of the rhodium system **Rh4** would not go through the usual direct [3 + 2]_C,O_ addition but through a two-step pathway on the singlet and triplet potential energy surfaces respectively. Frenking et al. had predicted that it would go through direct [3 + 2]_C,O_ addition with an activation barrier of −0.40 kcal/mol. However, on the doublet PES, a direct [3 + 2]_C,O_ addition pathway was located with an activation energy barrier of 3.17 kcal/mol which is the kinetically most favoured on all the potential energy surfaces. Unlike the iridium system, a direct addition through [3 + 2]_O,O_ is not located on the singlet PES, but a stepwise [1 + 1]_O,O_ addition between one oxygen of the complex with one carbon of ethylene to form the dioxylate. The pathway leading to the formation of such product **Rh3** on the triplet surface has overall barrier of about 24.75 kcal/mol and reaction energy of −19.27 kcal/mol whiles on the singlet PES (Fig. 4) the barrier and reaction energy are 49.45 and −205.19 kcal/mol respectively. Kinetically the reaction towards the formation of the species **Rh3** is favoured on the triplet PES but thermodynamically it is favoured on the singlet PES. Therefore there is the possibility of spin crossing from a triplet state transition state to a singlet state product. The [2 + 2] cycloaddition reaction across Rh=O leading to the formation of four-membered metallaoxetane **Rh8** (Fig. 4) has an activation barrier of 10.28 kcal/mol on the singlet PES and 25.92 kcal/mol on the doublet PES (Fig. 5). These barriers are lower than the barriers for the respective formation of the five-membered dioxylate complex through a stepwise [1 + 1]_O,O_ addition between one oxygen of the complex with one carbon of ethylene. The [2 + 2] cycloaddition reaction across Rh=C to form four-membered metalla complex **Rh10** (Fig. 6) on the triplet surface has an activation barrier of 11.98 kcal/mol and this is also less compared to the stepwise [1 + 1]_O,O_ addition between one oxygen of the complex with one carbon of ethylene to form the dioxelate which has a barrier of 37.28 kcal/mol. The addition across the Rh=O is located only on the singlet and doublet PESs and addition across the Rh=C is located on triplet PES only. The formation of species **Rh10** through [2 + 2] addition across the Rh=C which could not be located on the singlet PES is consistent with the earlier works of Haunschild and Frenking (2008a) but in this work the species was located on the triplet PES, an indication of possible spin crossing effect, likely from a singlet state reactant through a triplet transition state to a triplet product, with an activation barrier of 7.43 kcal/mol.

A transition state for direct addition was not located for the formation of the five-membered metallaoxetane and the dioxylate on the triplet PES, but a two-step pathway through a stepwise [1 + 1] addition involving the oxygen atom of the complex in the first step and the carbon atom of the complex in the second step to form the metallaoxetane and a stepwise [1 + 1]_O,O_ addition between one oxygen of the complex with one carbon of ethylene to form the dioxelate. The formation of the epoxide is found to have come from direct side on attack of C_2_H_4_ on one oxygen atom of **Rh1**. However, the epoxide in principle could arise from five pathways just as in the case of the iridium analogue. Only three of those pathways were located on the singlet and doublet PESs, and one on the triplet surface. The most favoured pathway towards the formation of the epoxide is the [1 + 2]_O_ addition on the doublet surface which has an energy barrier of 14.49 kcal/mol. The triplet PES yielded the usual [2 + 2] addition across the metal–carbon double bond unlike the triplet PES of the iridium system.

It is found that for the reaction of the RhO_2_(CH_2_)(CH_3_) complex, the [2 + 2]_Rh,O_ is the most plausible pathway on the singlet PES, the [3 + 2]_C,O_ is the most favoured pathway on the doublet surface, whiles the [2 + 2]_Rh,C_ is the most plausible pathway on the triplet PES. Generally, the formation of similar analogues have lower activation barriers for the Rh complexes than for the Ir complexes on all the surfaces explored, a trend consistent in the works of Aniayei et al. (2013a, b, c) and Ahmed et al. (2015a, b) in the catalytic oxidation of olefins and ketenes respectively using group VII transition metal oxides.

The order of the energetically most favorable addition reaction for the rhodium system on the singlet potential energy surface is found to be in the order [2 + 2]_Rh,O_ > s[1 + 1]_C,O_ > s[1 + 1]_O,O_. That on the doublet surface is [3 + 2]_C,O_ > [1 + 2] > [2 + 2]_Rh,O_ and the triplet surface is in the order [2 + 2]_Rh,C_ > s[1 + 1]_C,O_ > s[1 + 1]_O,O_.

### Reaction between CoO_2_(CH_2_)(CH_3_) and ethylene

**Fig. 7:**
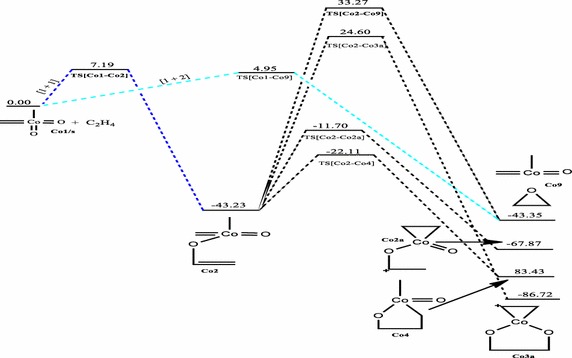
Energy profile of the reaction of CoO_2_(CH_2_)(CH_3_) with ethylene on the singlet PES at the B3LYP level of theory. Energies are in kcal/mol

**Fig. 8:**
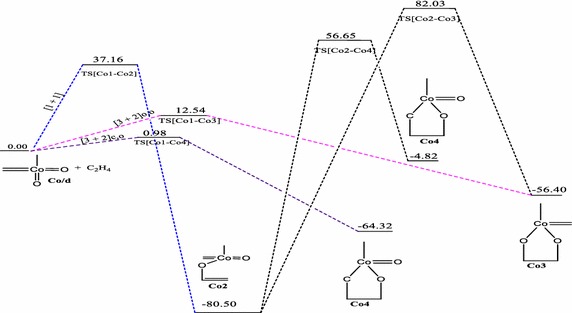
Energy profile of the reaction of CoO_2_(CH_2_)(CH_3_) with ethylene on the doublet PES at the B3LYP level of theory. Energies are in kcal/mol

**Fig. 9:**
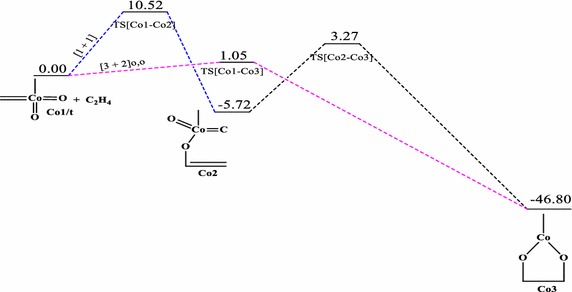
Energy profile of the reaction of CoO_2_(CH_2_)(CH_3_) with ethylene on the triplet PES at the B3LYP level of theory. Energies are in kcal/mol

The relative energies of the main stationary points involved in the reaction between CoO_2_(CH_2_)(CH_3_) with ethylene on the singlet, doublet and triplet PESs are shown on Figs. 7, 8, and 9 respectively.

A triplet Co(CH_2_)(CH_3_) reactant (**Co1/t**) has been computed to be 15.82 kcal/mol less stable in relation to the singlet structure **Co1/s**. All singlet and triplet structures were computed as neutral molecules whiles the doublet structures were computed as anions.

The reactions of **Co1** with ethylene does not form the typical [3 + 2] and [2 + 2] cycloaddition products in a concerted process on the singlet potential energy surface, a trend consistent with the earlier works of Haunschild and Frenking (2008a). However, a stepwise pathway was identified for the formation of the five-membered product in which such addition could have proceeded. However, on the doublet and triplet PESs (Figs. 8, 9), the usual [3 + 2] cycloaddition pathways were obtained; these were not reported in the earlier works (Haunschild and Frenking 2008a) due to the restriction of the work to only the singlet PES. The work by Haunschild and Frenking (2008a) could not exclude the fact that higher spin states may play a role in the reactions, especially for the cobalt species. The direct [1 + 2]_O_ addition yielding the epoxide homologue was obtained on the singlet state with activation barrier of 4.95 kcal/mol.

Activation barriers of 12.54 and 1.05 kcal/mol have been calculated for the [3 + 2]_O,O_ addition leading to the formation of the five-membered cycloadduct species **Co3** (Figs. 8, 9) on the doublet and triplet PES respectively with exothermicities of 56.40 and 46.80 kcal/mol respectively. A [3 + 2] cycloaddition across the C=Co=O of the metal complex to form species **Co4** (Fig. 8) on doublet PES has an activation energy of 0.98 kcal/mol and reaction energy of −4.82 kcal/mol. A stepwise pathway towards formation of the same species **Co4** is also identified on both the singlet and doublet PESs with overall activation barriers of 21.12 and 137.15 kcal/mol respectively. Formation of such product is kinetically and thermodynamically favoured on the singlet PES. The dioxylate species was localized on the triplet and doublet PESs through a stepwise addition process with overall activation barriers of 10.52 and 162.53 kcal/mol respectively. Formation of such product is kinetically favoured on the triplet surface but thermodynamically favoured on the doublet PES. The formation of the epoxide was located only on the singlet surface. Two pathways were identified for the formation of the epoxide on the singlet potential energy surface.

The most favourable pathway is the direct [3 + 2]_C,O_ addition with activation barrier of 0.98 kcal/mol on the doublet PES (Fig. 8) to form the five-membered intermediate **Co4.** The formation of the dioxylate species **Co3** on the triplet PES by [3 + 2]_O,O_ addition (Fig. 9) is highly competitive, with activation barrier of 1.05 kcal/mol. The formation of **Co3** and **Rh3** on all surfaces have lower activation barriers than the formation of **Ir3**, a trend consistent in the works of Aniayei et al. 2013a, b, c and Ahmed et al. (2015a, b) in the catalytic oxidation of olefins and ketenes respectively using group VII transition metal oxides. Again, the potential energy surfaces of the lower homologues have fewer competitive side reactions than those of the higher homologue, a similar trend was also noted in our earlier works (Aniayei et al. 2013a, b, c; Ahmed et al. 2015a, b) in the reactions of group VII metal complex with olefins and ketenes.

For the reaction of the CoO_2_(CH_2_)(CH_3_) complex, the [1 + 2]_O_ addition is the most plausible on the singlet PES, [3 + 2]_C,O_ cycloaddition to form the five–membered intermediate is the most preferred pathway on the doublet PES, whiles on the triplet PES the preferred pathway is the [3 + 2] addition across the O=Co=C bond of the metal complex to form the five-membered dioxylate intermediate.

### Comparison of the reactions of IrO_2_(CH_2_)(CH_3_), RhO_2_(CH_2_)(CH_3_) and CoO_2_(CH_2_)(CH_3_) with ethylene

Table 1 shows a comparison of the energetics of the first steps of the various reactions of IrO_2_(CH_2_)(CH_3_), RhO_2_(CH_2_)(CH_3_) and CoO_2_(CH_2_)(CH_3_) with ethylene on the singlet, doublet and triplet surfaces. It is seen from the table that the activation barriers for specific additions are lower for the CoO_2_(CH_2_)(CH_3_) than for RhO_2_(CH_2_)(CH_3_) and IrO_2_(CH_2_)(CH_3_). It is also seen that there are fewer competitive side reactions in the case of CoO_2_(CH_2_)(CH_3_) than for RhO_2_(CH_2_)(CH_3_) and IrO_2_(CH_2_)(CH_3_). This implies that CoO_2_(CH_2_)(CH_3_) may more efficiently and more selectively oxidize olefins to specific products than do RhO_2_(CH_2_)(CH_3_) and IrO_2_(CH_2_)(CH_3_).

With regard to the spin effects, it is seen that the activation barriers for the formation of the four—or five- membered metallacycle intermediates through [2 + 2] or [3 + 2] cyclo-addition are lower on the triplet PES than on the singlet PES for the formation of similar analogues. Also, there are fewer competitive reaction pathways on the triplet surface than on the singlet PES, a trend which is also found to be consistent with our earlier works (Aniayei et al. 2013a, b, c)

## Summary and conclusions

The following conclusions are drawn from the results presented.

1. In the reactions of the IrO_2_CH_2_CH_3_ complex, the most plausible pathways on the various surfaces are: singlet PES; [3 + 2]_C,O_ which is consistent with the work of Haunschild and Frenking (2008a), doublet PES; [3 + 2]_O,O_, triplet PES; stepwise [1 + 1] addition leading to the formation of the metallaoxetane.
2. The most favored reaction pathway for ethylene addition to the iridium system is the [3 + 2]_C,O_ yielding metallaoxetane as product on the singlet surface with activation barrier of 0.68 kcal/mol at the B3LYP level of theory. However, the triplet product is more stable than the singlet product. There is therefore the possibility of intersystem crossing from singlet reactant through singlet transition state to triplet metallaoxetane product, as illustrated in scheme 3b of the seminal work of Schröder et al. (2000) on two-state reactivity in organometallic chemistry.
3. The results for the rhodium system are similar to those of the iridium system. However, for the reaction of the RhO_2_(CH_2_)(CH_3_) complex, the [2 + 2]_Rh,O_ addition pathway is the most favoured on the singlet surface, the [2 + 2]_Rh,C_ is the most plausible pathway on the triplet PES and [3 + 2]_C,O_ is the most plausible on the doublet surface.
4. The most favored reaction pathway for ethylene addition to the rhodium system, kinetically and thermodynamically, is the [2 + 2]_Rh,O_ yielding four-membered metallaoxetane as product on the doublet surface, which has activation barrier and exothermicity of 3.17 and 75.54 kcal/mol respectively.
5. The rhodium system does not go through the usual direct [3 + 2]_C,O_ addition on the singlet PES as predicted by Haunschild and Frenking (2008a) with activation barrier of −0.40 kcal/mol, but would go through a stepwise [1 + 1] addition involving the oxygen atom of the complex in the first step and the carbon atom of the complex in the second step with an overall barrier of 23.87 kcal/mol but such addition is only possible on the doublet PES.
6. For the reaction of the CoO_2_CH_2_CH_3_ complex, the [1 + 2]_O_ addition is the most plausible on the singlet PES, [3 + 2]_C=Co=O_ cycloaddition to form the five–membered intermediate is the most preferred pathway on the doublet PES, whiles on the triplet PES the preferred pathway is the [3 + 2] addition across the O=Co=O bond of the metal complex to form the five-membered dioxylate intermediate.
7. The most favored reaction pathway for ethylene addition to the cobalt system is the [3 + 2]_C,O_ on the doublet PES with an activation barriers of 0.98 kcal/mol followed closely by [3 + 2]_O,O_ addition on the triplet PES with a barrier of 1.05 kcal/mol. The formation of the dioxylate analogue **Co3a** (Fig. 7) on the singlet PES is thermodynamically favoured with exothermicity of 86.72 kcal/mol.
8. The calculated activation and reaction energies for addition pathways at the MO6 level of theory are lower than those at the B3LYP, which is consistent with the work of Linder and Brinck (2012) and Ahmed et al. (2015a, b) but both levels predict the same preferred addition pathways and the same trends.
9. The reactions of olefins with the Co dioxo complex have lower activation barriers for the preferred [3 + 2] and [2 + 2] addition pathways as well as fewer side reactions than those of the rhodium and iridium. This trend was also seen in our earlier work with reactions of group VII metals with olefins (Aniayei et al. 2013a, b, c) and ketenes (Ahmed et al. 2015a, b). This could imply that the cobalt oxo complexes can efficiently and selectively catalyze specific reactions in oxidation of olefins than Rh and Ir oxo complexes will do and therefore Co oxo complexes may be better catalysts for specific oxidation reactions of olefins than Rh and Ir complexes are.
10. The activation barriers for the formation of the four—or five- membered metallacycle intermediates through [2 + 2] or [3 + 2] cyclo-addition are lower on the triplet PES than on the singlet PES for the formation of similar analogues. There are fewer competitive reaction pathways on the triplet surface than on the singlet PES. Also, cycloadditions that seem impossible on the singlet PES seem possible on the doublet and or triplet PESs, this is the case typically for the Rh and Co complexes. These findings illustrate the importance of multiple spin states in organometallic reactions.
